# A European project on incidence, treatment, and outcome of sarcoma

**DOI:** 10.1186/1471-2458-10-188

**Published:** 2010-04-12

**Authors:** Giuseppe Mastrangelo, Emanuela Fadda, Luca Cegolon, Maria C Montesco, Isabel Ray-Coquard, Alessandra Buja, Ugo Fedeli, Alvise Frasson, Paolo Spolaore, Carlo R Rossi

**Affiliations:** 1 Department of Environmental Medicine and Public Health, University of Padua, Padua, Italy; 2 Section of Pathology, Department of Oncology and Surgery, University of Padua, Padua, Italy; 3 Centre Léon-Bérard, 28 Rue Laënnec, 69373 Lyon, France; 4 SER - Regional Epidemiological Service, Veneto Region, Castelfranco Veneto, Italy; 5 Surgery Branch, Department of Oncological and Surgical Sciences, University of Padua, Padua, Italy

## Abstract

**Background:**

Sarcomas are rare tumors (1-2% of all cancers) of mesenchymal origin that may develop in soft tissues and viscera. Since the International Classification of Disease (ICD) attributes visceral sarcomas (VS) to the organ of origin, the incidence of sarcoma is grossly underestimated. The rarity of the disease and the variety of histological types (more than 70) or locations account for the difficulty in acquiring sufficient personal experience. In view of the above the European Commission funded the project called Connective Tissues Cancers Network (CONTICANET), to improve the prognosis of sarcoma patients by increasing the level of standardization of diagnostic and therapeutic procedures through a multicentre collaboration.

**Methods/Design:**

Two protocols of epidemiological researches are here presented. The first investigation aims to build the population-based incidence of sarcoma in a two-year period, using the new 2002 WHO classification and the "second opinion" given by an expert regional pathologist on the initial diagnosis by a local pathologist. A three to five year survival rate will also be determined. Pathology reports and clinical records will be the sources of information.

The second study aims to compare the effects on survival or relapse-free period - allowing for histological subtypes, clinical stage, primary site, age and gender - when the disease was treated or not according to the clinical practice guidelines (CPGs).

**Discussion:**

Within CONTICANET, each group was asked to design a particular study on a specific objective, the partners of the network being free to accept or not the proposed protocol. The first protocol was accepted by the other researchers, therefore the incidence of sarcoma will be assessed in three European regions, Rhone-Alpes and Aquitaine (France) and Veneto (Italy), where the geographic distribution of sarcoma will be compared after taking into account age and gender. The conformity of the clinical practice with the recommended guidelines will be investigated in a French (Rhone Alps) and Italian (Veneto) region since the CPGs were similar in both areas.

## Background

### Epidemiological challenges of sarcoma

Sarcomas are rare tumors (1-2% of all cancers) originating from connective tissue, skin, retroperitoneum, bone, and viscera; Kaposi's sarcoma is usually excluded or reported apart. In the Venetian Tumour Registry (VTR), the incidence data (per 100,000) from 2000 to 2004 for sarcomas are shown in Table 1.

**Table 1:** Incidence data (per 100,000) for sarcomas, 2000-2004.

	N. of cases	Freq. %	Crude rate	Age stand. rate	
				Europe	World
Soft tissue sarcoma (males)	180	0.6	3.9	3.3	2.6
Kaposi's skin (males)	48	0.1	1.0	0.8	0.6
Soft tissue sarcoma (females)	136	0.5	2.8	2.2	1.8
Kaposi's skin (females)	15	0.1	0.3	0.1	0.1

Data from the Veneto Tumor Registry (VTR).

Tumor registry data derive from a cross-check among three different sources: biopsy records of pathology departments, hospital discharge forms, and death certificates. The latter sources use the International Classification of Disease (ICD) that attributes visceral sarcomas (VS) to the organ of origin. The incidence of sarcomas is thus grossly underestimated.

Tumor classifications have become an integral part of modern oncology and provide pathologists with guidelines to facilitate diagnostic and prognostic reproducibility.

In late 2002, the World Health Organization (WHO) published a new classification of sarcoma. Since it represents a broad consensus view, it has gained widespread acceptance. The four most significant conceptual advances have been: (i) the formal recognition that morphologically benign lesions (such as cutaneous fibrous histiocytoma) may very rarely metastasize; (ii) the general acceptance that most pleomorphic sarcomas can be meaningfully sub-classified and that the so-called malignant fibrous histiocytoma is not a definable entity, but instead represents a wastebasket of undifferentiated pleomorphic sarcomas, accounting for no more than 5% of adult soft tissue sarcomas (STS); (iii) the acknowledgement that most lesions formerly known as haemangiopericytoma show no evidence of pericytic differentiation and, instead, are fibroblastic in nature and form a morphological continuum with solitary fibrous tumors; and (iv) the increasing appreciation that the derivative cell type(s) of most STS (histogenesis) is unknown and, hence, an increasing number of tumors are placed in the "uncertain differentiation" category [1].

To our knowledge, there are few studies that have reported morphology-specific tabulations of sarcoma, using the 2002 WHO classification criteria. Toro et al. [2] analyzed the 1978-2001 Surveillance, Epidemiology and End Results program incidence rates of sarcoma regardless of primary site, except from bones and joints, using the 2002 WHO classification criteria. There were 26,758 cases available for the analysis. Almost half (47.9%) of all sarcomas developed in soft tissues, 14.0% in the skin and 7.0% in the uterus. Overall, the incidence rates were highest among black women (6.26/100,000 person-years) and lowest among white women (4.60/100,000). Age adjusted rates increased at 1.2% and 0.8% per calendar year among white males and females, respectively, both trends being statistically significant, while rates among blacks declined slightly. Total sarcoma rates rose exponentially with age. Rates for both uterine leiomyosarcoma and dermatofibrosarcoma increased rapidly during the childbearing years, peaking at about the age of 40 and 50 respectively. The incidence patterns of sarcoma varied markedly by histological type, underpinning the notion that these tumors may be etiologically distinct.

### Diagnostic challenges

Although sarcomas are rare cancers, they include more than 70 different histological subtypes. Furthermore, difference in diagnostic criteria as well as the use of new techniques (i.e. immunohistochemistry, rt-PCR, molecular genetics, genomics) complicate their histotype definition.

There is a considerable subjective variability among physicians in making observations on even the simplest of events. For instance, in clinical studies involving assessment of imaging methods like X-Rays, ultrasounds, and CT scans, the value of the study can be better assessed if measures of variability are evaluated and reported. Histological examinations are also imaging techniques. When it comes to cancer, a pathological diagnosis is the "gold standard" that indicates the presence or absence of the malignancy, the type of cancer, and its classification. Due to the fact that therapeutic decisions are based on the presumed reliability of the pathology diagnosis, a misdiagnosis can result in unnecessary, harmful and aggressive therapy or inadequate treatment. Unfortunately, medical studies over the last two decades have demonstrated that this gold standard is not consistently reliable for sarcoma. In fact, multiple studies have demonstrated discrepancy rates of up to 30% with an average of approximately 10%. A "discrepancy" happens when one pathologist renders a diagnosis and another pathologist looks at the same material and provides a different opinion [3]. For example, a major discrepancy was found in 25% of 266 cases of soft tissue lesions reviewed by an expert. Of these discrepancies, 45% consisted of benign lesions diagnosed as sarcomas, and 23% were sarcomas diagnosed as benign tumors [4]. Obtaining a wrong pathological report is not limited to the U.S. Other countries have found similar problems. For example, in the United Kingdom, 413 cases of sarcoma were reviewed and the diagnosis was confirmed in only 76% of instances. The study concluded that "second opinion is essential in cases of presumed sarcoma ... to ensure that appropriate treatment is selected." [5].

In order to analyse the impact of modern sarcoma classification criteria, Daugaard [6] reviewed the pathological material from 281 extremity STS. The cases were originally diagnosed between 1972 and 1994, and the most frequent diagnoses were then malignant fibrous histiocytoma (MFH) (26%), liposarcoma (21%), fibrosarcoma (11%), and leiomyosarcoma (10%). After reclassification, the proportions had changed significantly, with the largest group now being leiomyosarcomas (20%), liposarcomas (17%), synovial sarcomas (14%), and sarcomas "Not Otherwise Specified" (NOS) (11%). The original diagnosis was changed in 57% of the cases; in particular, the number of fibrosarcomas was reduced from 32 to 6, and MFHs from 72 to 2, with 22 renamed as myxofibrosarcomas; 20 (7%) were found not to be sarcomas.

The vast majority of pathologists' diagnoses are correct. However, a minority of cases benefit from a second opinion. One factor to consider is how rare the cancer is. If it is rare, chances are that a pathologist has not seen many of this type of tumor. Although the overall percentage of affected cases is not large, the consistent rate of discrepant diagnosis uncovered by second opinion surgical pathology may have an enormous human and financial impact. Accordingly, the authors recommend that review of the original histological material should be undertaken prior to the institution of a major therapeutic endeavour [7].

### Therapeutic challenges

Quality of care is defined by WHO as "a process that allows one to guarantee to each patient the range of diagnostic and therapeutic procedure that will ensure the best outcome in terms of health in conformity with Evidence-Based Medicine, in the most costs effective manner, at the lesser iatrogenic risk and for the highest satisfaction in terms of procedure for the patient, outcomes and human relationships within the health care system". According to [8], this process involves an evaluation of:

• structure (characteristics of the care providers, their tools and resources, and the physical/organizational setting);

• process (both interpersonal and technical aspects of the treatment process);

• outcome (change in the patient's symptoms and functioning).

Geographic variation of medical procedure utilization is a well known issue in industrialized countries [9-14]. The characteristics of hospital structures could be responsible for variations in practices, sometimes inducing large differences in the consumption of resources [15]. Another factor is the big size of the institution which seems to promote the use of more sophisticated techniques [16]. The determinants of medical decision-making remain to be evaluated and specified. Since patients may receive care which results in adverse rather than beneficial results with more adverse than beneficial effects, professional practice variations represent an essential area of research.

In order to prove the relationship between differences in mortality and differences in medical practice or medical organisations, a suitable observational ground seems to be the study of rare tumours, such as sarcomas. The rarity of the disease, the variety of histological types or locations and the heterogeneity of prognostic factors associated with local or distant spread, account for the difficulty in acquiring sufficient personal experience. Moreover, the lack of graduate or post-graduate medical teaching makes it difficult for physicians outside centres of excellence to provide appropriate sarcoma management. Every stage of the treatment remains controversial, and this is why multidisciplinary co-ordination is essential. A number of scientific publications have suggested the existence of a causal link between medical practices and patient outcome [17]. In France (Rhone-Alpes region), a retrospective analysis of the medical records of 100 patients with localized STS (stage TxNxM0) revealed significant correlations between:

• local relapse and: quality of resection (p = .01); macroscopic extent of surgery (p = .002); subspecialty of the surgeon (p = .03); type of hospital (p = .02); review of patient file by multidisciplinary committee before surgery (p = .02); management in the cancer network (p = .02); conformity of radiotherapy with clinical practice guidelines (CPGs) (p = .007);

• metastatic relapse and: histological grade (p = .008); multidisciplinary committee decision after surgery (p = 0.04)]; and management in the cancer network (p = .02) [18].

The retrospective nature of this study did not appear sufficient to ensure a satisfactory conclusion; thus a prospective study design will be adopted, and investigation will be conducted on a larger scale in order to determine the impact of each prognostic factor on patient survival.

### Research objectives

In view of all the above, the European Commission has recently approved the project CONnective TIssues CAncers NETwork (CONTICANET), which aims to improve the prognosis of sarcoma patients by increasing the level of standardisation of diagnostic and therapeutic procedures (Additional File 1). The study received formal approval by the Ethical Committee of the Padua University Hospital.

Within the frame of the CONTICANET project, we will collect prospectively a population-based series of STS and VS cases in the Veneto Region during 2007 and 2008 (two years), with the aim to:

• build up the population-based incidence of sarcoma in a two-year period (2007-2008), using the new WHO classification of 2002 and a "second opinion" given by an expert regional pathologist about the diagnosis of a local pathologist. The three to five year survival period will also be ascertained (Study 1);

• the second study aims to compare in patients with STS, VS, and gastro-intestinal stromal tumors (GIST, a histological type of VS) the effects on survival or relapse-free period - allowing for histological subtypes, stage, primary site, age and gender - when the disease was treated or not in compliance with CPGs (Study 2).

## Methods/Design

### Study 1. Estimating the population-based incidence of sarcoma (2007-2008), the three- to five-year survival rate, and the causes of death

Activities undertaken before the date of issue of the present protocol are described by using passive voice and past tense. The active voice and present (or future) tense is used for any repetitive technical activity, as well as for any administrative procedure, that is being followed in order to achieve the study objectives.

#### Case definition

Cases will be subjects:

• of both genders and any age, residing in Veneto;

• with histologically proven malignant sarcoma (classification in Additional File 2), diagnosed as a first cancer during 2007 and 2008, (despite the fact that distant metastasis were present at initial diagnosis).

Osteosarcoma, sarcomatoid carcinoma, mesothelioma, neuroblastoma, paraganglioma, and mixed (epithelial and mesenchymal) tumors of the female genital tract will be excluded, as well as patients (any histology) with a relapse of sarcoma in the observation period.

#### Scrutinizing the pathology network

The principal source of cases will be the regional network of pathologists. Three expert leaders for second opinion on sarcoma in Veneto agreed to support the study.

##### BARRIERS

In order to achieve the study objective, all the regional Pathology Departments were identified and the following barriers were overcome:

• lack of cooperation;

• obstacles due to confidentiality and internal rules;

• type of data storage and data processing;

• type of morphological classification and codes for case classification;

• procedures for case identification and sorting.

*Lack of cooperation*

All the data needed were located in the pathology records. In an automated environment, reporting could be as simple as a few keystrokes. Unfortunately, in Veneto an automated reporting of pathological diagnoses to a single archive does not exist, thus rendering our study heavily dependent on the actions of very busy pathologists. Therefore, the pathologists of Veneto were personally invited to team up with the expert leaders to whom they apply for the second opinion for sarcoma. A person was assigned to clinical monitoring in order to help the collection of cases from the Pathology departments every three months.

*Obstacles by confidentiality legislation and internal rules*

The clearance from the Ethical Committee was obtained along with the support of the managing tams of all the Local Health Units (LHUs) in Veneto. An informed consent will be requested from all patients for data storage and follow-up.

*Type of morphological classification and codes for cases classification*

All pathologists agreed to use the morphological classification of WHO in 2002 [19] and the SNOMED codes for sarcoma in their daily practice. Classification and codes are reported in Additional File 2.

*Procedures for cases identification and sorting*

In the pathology departments of Veneto, computerized data are processed by using different software packages: Armonia, WindoPath, APSIS, and others. All departments in Veneto have to be scrutinized.

*Second opinion diagnosis*

The Section of Pathology (Department of Oncology and Surgery, University of Padua) and the Department of Pathology (Treviso Regional Hospital), perform both primary cancer diagnosis and second opinion diagnosis. The primary diagnoses of Padua will be reviewed by Treviso experts and vice versa.

*Scrutinising other archives*

As already known, the incident cases of sarcoma will be searched through parallel scrutiny of multiple independent sources: network of pathologists and archives of hospital discharges.

##### Archive of hospital discharge records

The archive of hospital discharge forms will be scrutinised through a coordinated action with the Regional Epidemiological Service of the Veneto Region (SER). All admissions with ICD-9 codes 171, 158 and 176 among discharge diagnoses (all diagnostic positions) will be extracted four times/year from the electronic archives of hospital discharge records available at SER. Repeated admissions of the same subject will be identified, and prevalent cases (subjects hospitalised with the relevant diagnoses in 2005-2006) will be excluded. The list of subjects identified by discharge records will be sent to the clinical monitor to ascertain the diagnosis in those not retrieved through the other sources. If the patient was not already known, the clinical monitor will report the information forms to the physician in charge of the epidemiological study.

##### Tracing clinical records

All medical records regarding the above patients will be traced and photocopied from the relevant hospital offices.

*Statistical analysis*

The incidence will be calculated by age, gender, main nosological group (STS and VS), primary site and the histological type of the tumor. The incidence rates will be age-standardised with the direct method, using the World and European populations as standard. Survival rates will be calculated by the Kaplan-Meier method [20]. A capture-recapture approach will be used to determine and improve accuracy in estimating frequency of STS and VS [21].

### Study 2. Evaluation of medical practices and their impact on the management of a rare malignant tumor: an application for the management of sarcoma in the Veneto region

#### Case definition

We will include patients:

• of both genders;

• residing in Veneto;

• older than 18 years at initial diagnosis;

• with histologically proven STS, VS, or GIST, diagnosed and/or treated from 2007 to 2008, without distant metastasis at initial diagnosis. Only cases of localized or locally advanced STS, VS, or GIST will be selected (disease stage: TxNxM0).

We will exclude:

• patients seen for a relapse of sarcoma in the observation period;

• patients whose treatment had not been managed in Veneto.

#### Study size

In the study by [18], cases of local relapse after treatment were 22% (= 9/39) or 44% (= 27/61) in sarcoma patients with or without multidisciplinary committee before/after surgery respectively (Table 2).

**Table 2:** cases of local relapse after treatment in sarcoma patients with or without multidisciplinary committee before/after surgery.

		Local relapse		Total
		Yes	No	
Multidisciplinary committee before/after surgery	Yes	9	30	39
	No	27	34	61

Therefore, the computation of the study size assumes that:

• the differences between the two proportions (namely 22% and 44%) corresponded to the expected effect;

• the significance criterion (alpha) is set at 0.05;

• the test is 2-tailed, which means that an effect in either direction will be interpreted.

Based on these assumptions, the study should include 200 (or 150) patients in order to have a 89% (or 78%) power to yield a statistically significant result.

#### Collection of clinical data

Surgeons, oncologists and pathologists will be approached (in ad hoc meetings) for consent to produce hospital records of patients under their care for the purpose of this study.

All medical records of each patient will be photocopied, and data on clinical history and treatment will be collected from medical records during the course of the study. Information to be collected on a standardised form includes:

• registry data of the patient, identification of hospital and physicians in charge;

• clinical features, imaging studies, stage of the disease;

• date and methods of the initial diagnosis (before surgery), histology of surgical specimen and histology of metastases;

• description of the initial surgical operation, particular techniques (limb perfusion, brachitherapy), surgical removal of metastases;

• radiotherapy: characteristics and response;

• chemotherapy: characteristics and response;

• relapse: date, diagnosis, and topography;

• surgical intervention after relapse including particular techniques and removal of metastases (histology of metastases);

• radiotherapy after relapse: characteristics and response; chemotherapy after relapse: characteristics and response;

• patients follow-up and eventual date and cause of death;

• analysis of the process: compliance with guidelines; multidisciplinary coordination at different steps (before biopsy, before surgery, before adjuvant therapy, at first and following relapses).

#### Compliance with Clinical Practice Guidelines (CPGs)

While other sections of the above form will be completed by a single clinical research assistant, the last section of the questionnaire (regarding compliance) will be collected by a single investigator. Observations made by a single observer will avoid inter-observer reproducibility biases. Reproducibility in the analysis of decisions across patients will be evaluated during the course of the study by control from pre-selected experts and by reassessment of random records from the same expert blinded to the results of the previous analysis.

The CPGs is a nationwide project of the Italian National Research Council (CNR). The first volume of the standards, option and recommendations (SOR) for the clinical management of cancer was published in 2003 [22]. These SOR were accepted with minor discrepancy by the oncologists of the Veneto Region. The clinical final release was diffused in 2004.

##### Criteria for optimal initial examination and diagnosis

(i) Clinical size and depth of the mass must be recorded; (ii) computed tomography (CT) is required for abdominal localization, or magnetic resonance imaging (MRI) of the mass for limb localization; (iii) chest radiograph or CT scan is required to identify metastases; and (iv) pre-operative biopsy (incisional or needle) should be performed, preferably by the surgeon in charge of future surgical management. Exception is made for small tumors <3 cm, where excisional biopsy is accepted.

##### Criteria for optimal surgical management

Whenever possible, primary surgery should perform wide excision with 1-2 cm margins. For high-grade lesions, large (>3 cm) tumors, or deep-seated tumors, surgery alone is acceptable only in case of amputation or compartimental resection with negative histological margins (R0). Wide excision alone, with no adjuvant treatment, is acceptable only for superficial, small (<3 cm) and low-grade lesions. Histologically positive margins (R1) or incomplete excision (R2) have to be considered inadequate, and should be followed by further appropriate treatment (further surgery or adjuvant therapy if formal review by a multidisciplinary sarcoma committee considers surgery non-feasible). TNF-based isolated limb perfusion can be proposed as neo-adjuvant treatment in patients candidates for amputation or as palliative care.

##### Criteria for histopathological report

The following parameters should be reported: i) histological type (according to WHO system); ii) histological grade; iii) size of the tumor; iii) status of resection margins (minimum distance); iv) results of ancillary studies (immuno-histochemical, cytogenetic and molecular studies).

##### Criteria for optimal radiation therapy management

Association between wide excision and adjuvant radiation therapy should be considered the gold standard treatment. The absence of adjuvant radiotherapy is acceptable for superficial, small (<3 cm) and low-grade tumors, and for limb sarcomas when amputation is performed. For non-operable sarcomas, primary radiation therapy could be an option. Optimal technical criteria are: (i) 50 Gy delivered dose [with an additional boost of 10 Gy in case of microscopic residual tumor (R1)]; (ii) target volume to irradiate encompassing tumor bed, scars, including draining orifices, with appropriate security margins; and (iii) delay from surgery to radiation therapy not exceeding 12 weeks.

##### Criteria for optimal chemotherapy management

For non-readily, non-operable sarcomas, primary chemotherapy can be an option, as can radiation therapy. For readily operable sarcomas, neo-adjuvant chemotherapy should be performed only as part of a clinical research protocol. In the adjuvant setting, systemic chemotherapy should be performed only within the context of a prospective clinical trial. Adjuvant chemotherapy could be performed for patients with histologically positive margins after wide surgery.

##### Criteria for optimal post-therapeutic surveillance

There is no consensus concerning the surveillance of sarcomas. Recommendations of the SOR are follow-up with clinical examination every 12 weeks during the first 3 years, then every 6 months until the fifth year post-management. Clinical examination and chest radiograph or CT scan and ultrasound examination are the prevailing tools to identify metastases.

#### Statistical analysis

Overall survival will be measured from the date of diagnosis to the date of death or to the day of loss to follow-up for living patients. Relapse-free survival is defined as the interval between curative treatment and diagnosis of relapse. Stable disease is defined as the documented presence of a non respectable tumor or residual tumor after surgery. For these patients with stable disease, the estimated relapse-free survival is 0 months. The analyses of patterns of care and survival will be restricted to STS and VS as a whole, or GIST.

Treatment centres (identified from the SER's records) will be classified as follows:

• teaching hospitals attached to medical schools;

• non-teaching hospitals: the remaining regional (Veneto) hospitals treating study patients.

Descriptive statistics such as absolute and relative frequencies, mean and standard deviations (range), as well as χ^2^-test, Fisher's exact test, Student's t-test, ANOVA or Mann-Whitney U-test will be performed where appropriate. Time to progression and survival will be analyzed using the Kaplan-Meier method, together with the log rank test [20]. For a multivariable approach, Cox proportional hazard regression analysis will be used. A multivariable logistic regression analysis will be used to identify predictors of adequacy of clinical decisions with Evidence-Based Medicine. The analysis will take into account the fitting of the following factors: institution, physician and patient. In all statistical analyses p < 0.05 will be considered to indicate statistical significance.

## Discussion

Within the CONTICANET network there were nine main activities or work packages (WP): Network Management (WP1); Network sustainability (WP2); Network methodologies and infrastructures (WP3); Epidemiology (WP4); Molecular characterization (WP5); Target characterization, drug screening and non-clinical pharmaco-dynamics (WP6); Clinical pharmacology, clinical pharmaco-dynamics and pharmaco-vigilance (WP7A); Regional therapy, including surgery and radiotherapy (WP7B); Dissemination (WP8); and Transfer of Excellence (WP9). Moreover, within each WP there were several tasks and task groups. For example WP4 (Epidemiology) included the following research activities:

• Descriptive epidemiology;

• Molecular epidemiology;

• Determinants of medical practice;

• Education directed to medical practitioners, surgeons and pathologists.

Each group was asked to design a particular study on a specific objective, the partners of the network being free to accept or not the proposed protocol.

The protocol of the above Study 1 (incidence of soft tissue and visceral sarcoma) was proposed by the University of Padua and accepted by two other partners. Therefore, the incidence and survival of sarcoma will be ascertained in three European regions (two French regions: Rhone-Alpes and Aquitaine and one Italian region: Veneto). In a collateral future study, the geographic distribution of sarcoma according to histotype (and adjusting for age and gender) will be compared between and within the regions. Clustering of patients with sarcomas could allow the establishment of hypotheses on risk factors underpinning the particular spatial distributions of cases. Subsequently, an ecological study will be planned to investigate whether environmental risk factors could affect the incidence of sarcoma. Lastly, a case-control study will be designed to assess the risk factors for soft tissue and visceral sarcoma and the major histological types.

The protocol of the above Study 2 (compliance of medical care with the CPGs for sarcoma) was accepted by another partner. Therefore, conformity of clinical practice with the CPGs will be investigated in France (Rhone Alps Region) and Italy (Veneto Region) since French and Italian guidelines are similar. A collateral new study could be planned on the cost-effectiveness analysis of compliance with CPGs for sarcoma management, during the whole clinical pathway of patients from diagnosis to death or end of follow-up. The analysis of these relations could be useful to advise public health policy makers on the economic consequences of CPGs for rare tumors, within a long term perspective.

## Abbreviations

CNR: National Research Council (Italian Acronym); CPGs: Clinical Practice Guidelines; CONTICANET: CONnective TIssue CAncers Network; GIST: Gastro Intestinal Stromal Tumor; ICD: International Classification of Diseases; LHU: Local Health Unit; SER: Regional Epidemiological Service of the Veneto Region (Italy); SNOMED: Systematized Nomenclature of Medicine; SOR: Standards, Option and Recommendations; STS: Soft Tissue Sarcoma; VS: Visceral Sarcoma; VTR: Venetian Tumor Registry; WHO: World Health Organization.

## Competing interests

The authors declare that they have no competing interests.

## Authors' contributions

GM and LC participated in the design of the study and the drafting of the paper; EF contributed in the drafting of the paper, UF, AB and PS provided epidemiological advice, MCM provided pathological expertise; AF and IRC provided technical medical advice; CRC coordinated the project and is the guarantor. All authors read and approved the final manuscript.

## Pre-publication history

The pre-publication history for this paper can be accessed here:

http://www.biomedcentral.com/1471-2458/10/188/prepub

## Supplementary Material

Additional file 1 Research Contract by the Commission of the European Communities (Research Directorate General). Click here for file

Additional file 2 WHO Classification of Sarcomas. Click here for file
