# Supplementary material for: A European project on incidence, treatment, and outcome of sarcoma
Source: BMC Public Health. 2010 Apr 12;10:188. doi: 10.1186/1471-2458-10-188 (PMC2882909; doi:10.1186/1471-2458-10-188)
Supplement: Additional file 1 — Research Contract by the Commission of the European Communities (Research Directorate General). [file 1471-2458-10-188-S1.PDF]

COMMISSION OF THE EUROPEAN COMMUNITIES

RESEARCH DIRECTORATE-GENERAL

Integrating and strengthening the European Research Area

Network of Excellence

CONTICANET

CONnective TIssue CAncers NETwork to integrate European Experience in Adults and Children

LSHC-CT-2005-018806

Contract Number 018806

CONTRACT No 018806 (LSHC-CT-2005-018806)

Network of Excellence

The **European Community** (the "*Community*"), represented by the **Commission of the European Communities** (the "*Commission*"), itself represented for the signature of this *contract* by Achilleas Mitsos, Director General for Research Directorate-General or his duly authorised representative,

of the **one part,**

and Université Claude Bernard Lyon 1, established in 43 bd du 11 novembre 1918, Lyon, 69622, France, represented by Domitien DEBOUZIE, President, and/or Jean-François MORNEX, Vice-President of the Scientific Board, or her/his/their authorised representative the *contractor* acting as *coordinator* of the *consortium*, (the "*coordinator*") and the other *contractors* identified in Article 1.2 below,

of the **other part**

HAVE AGREED to the following terms and conditions established in this contract and its annexes (the "*contract* ").

Article 1 - Scope

1. The *Community* agrees to grant a financial contribution for the implementation of a *project* called *CONnective TIssue CAncers NETwork to integrate European Experience in Adults and Children (CONTICANET)* within the framework of the specific research and technological development programme "Integrating and strengthening the European Research Area" (the "specific programme").

2. The *consortium* is composed of the *contractor* acting as *coordinator* and the following legal entities, who shall accede to the *contract* in accordance with the procedure referred to in Article 2, as *contractors* assuming the rights and obligations established by the *contract* with effect from the date on which it enters into force:

- **Allgemeines Krankenhaus St Georg,** established in Lohmühlenstr. 5, Hamburg, 20099, Germany represented by Axel-Rainer Hanauske, Chair, Chief, and/or Achim Gaessler, Director, or her/his/their authorised representative ("*contractor*")
- **National University of Ireland, Cork - University College Cork,** established in Western Road, Cork, Ireland represented by Robin Graham, Secretary and chief legal officer, and/or Michael Farrell, Assistant Secretary, or her/his/their authorised representative ("*contractor*")
- **Erasmus Medisch Centrum,** established in Dr.Molenwaterplein 40, Rotterdam, 2040, 3000 CA, Netherlands represented by Gerrit Stoter, Department Head, and/or Jacob Verweij, Department Deputy-head, or her/his/their authorised representative ("*contractor*")
- **The Institute of Cancer Research: Royal Cancer Hospital,** established in 123 Old Brompton Road, London, SW7 3RP, Great Britan represented by Andrew Whitehead, Director of Finance, and/or David Begg, Senior Management Accountant, or her/his/their authorised representative ("*contractor*")
- **Institut Gustave Roussy,** established in 39 rue Camille Desmoulins, Villejuif, 94805, France represented by Thomas Tursz, Director, and/or Bertrand Martin, Vice-director, or her/his/their authorised representative ("*contractor*")
- **Istituto Nazionale per lo Studio e la Cura dei Tumori,** established in Via G. Venezian 1, Milano, 20133, Italy represented by LOREDANA MASPES, High Commissioner, or her authorised representative ("*contractor*")

• **Ipsogen SAS**, established in Luminy Biotech Entreprises, Case 923, 163 Avenue de Luminy, Marseille, 9, 13009, France represented by Vincent FERT, CEO, and/or Stéphane DEBONO, COO, or her/his/their authorised representative ("*contractor*")

• **Ecole Normale Supérieure de Cachan**, established in 61, avenue du Président Wilson, Cachan, 94235, France represented by Claire Dupas, Director, and/or Hisham ABOU-KANDIL, Deputy Director, or her/his/their authorised representative ("*contractor*")

• **Institute of Oncology Ljubljana**, established in Zaloska 2, Ljubljana, 1000, Slovenia represented by Zvonimir Rudolf, Director general, or his authorised representative ("*contractor*")

• **PharmaMar Sociedad Anonima Sociedad Unipersonal**, established in Poligono Industrial La Mina, Avda. de los Reyes, 1, Colmenar Viejo, Madrid, 28770, Spain represented by Isabel Lozano, Chief Executive Officer, and/or Luis Mora, Chief Financial Officer, or her/his/their authorised representative ("*contractor*")

• **Sanofi-Aventis**, established in 174, avenue de France, Paris, 13, 75635, France represented by Gérard LE FUR, Scientific and Medical Director, or his authorised representative ("*contractor*")

• **Università Degli Studi di Padova**, established in Via VIII Febbraio , Padova, 35128, Italy represented by Mario Lise, Director, and/or Alberto Amadori, Vice-director, or her/his/their authorised representative ("*contractor*")

• **Katholieke Universiteit Leuven**, established in Oude Markt 13, Leuven, 3000, Belgium represented by Koen Debackere, Managing Director, and/or Paul Van Dun, Director, or her/his/their authorised representative ("*contractor*")

• **Centre Hospitalo-universitaire Saint Louis**, established in 1, avenue Claude Vellefaux, Paris, 10, 75475, France represented by Jean-Patrick Lajonchère, Chief executive manager, and/or Christian Nicolas, Assistant chief executive, or her/his/their authorised representative ("*contractor*")

• **ARTTIC**, established in 58 A rue du Dessous des Berges, Paris, 75013, France represented by Eric Papon, Managing Director, or his authorised representative ("*contractor*")

• **EZUS LYON 1**, established in 43 bd du 11 novembre 1918 - Bâtiment l'Atrium, Villeurbanne, BP 2107, 69616, France represented by Eric BERTHOD, Chairman of the Board of Directors, and/or Gérard POSA, General manager, or her/his/their authorised representative ("*contractor*")

• **Institut Bergonié**, established in 229, Cours de l'Argonne, Bordeaux, 33076, France represented by Josy REIFFERS, Director, and/or Christian FILLATREAU, General secretaty, or her/his/their authorised representative ("*contractor*")

• **Institut Curie**, established in Rue d'Ulm, Paris, 05, 75248, France represented by Claude Huriet, President, and/or Anne Bellod, General Secretary, or her/his/their authorised representative ("*contractor*")

• **Ruprecht-Karls-Universität Heidelberg**, established in Seminarstr.2, Heidelberg, 69117, Germany represented by Marina Frost, Chancellor, and/or Norbert Huber, EC Research officer, or her/his/their authorised representative ("*contractor*")

(hereinafter referred to as the "*contractors* ").

3. The *consortium* shall carry out the work set out in Annex I to this *contract* (the "*project*") up to the milestone specified in Annex I in accordance with the conditions set out in this *contract*.

4. The *contractors* are deemed to have concluded a *consortium agreement* regarding the internal operation and management of the *consortium*. *The consortium agreement* shall include all aspects necessary for the management of the *consortium* and the implementation of the *project* as well as any necessary intellectual property provisions.

Article 2 - Constitution of the *consortium*

1. The *coordinator* shall ensure that the legal entities identified in Article 1.2 complete the formalities

for them to accede to the *contract*. At the latest 60 calendar days after the entry into force of the *contract*, the *coordinator* shall send to the *Commission* one of the three duly completed and signed originals of Form A (set out in Annex IV), which shall be obtained from each of the *contractors* identified in Article 1.2. The two remaining signed originals shall be kept by the *coordinator* and the *contractor* concerned and be made available for consultation at the request of any other *contractor*.

2. Should any legal entity identified in Article 1.2 fail or refuse to accede to the *contract* within the deadline established in the previous paragraph, the *Commission* is no longer bound by its offer to contract with the said legal entity(ies). The *Commission* may terminate the *contract* in accordance with Article II.15.5, where any legal entity identified in Article 1.2 does not accede to the *contract* in accordance with the provisions established by the *Commission*.

3. However, the *consortium* may propose appropriate solutions to the *Commission* to ensure the implementation of the *project* including, where necessary, the accession to the *contract* of legal entities other than those identified in Article 1.2 in accordance with the provisions in Article 3.

4. In the case of termination, no costs incurred by the *consortium* under the *project* up to the date of *contract* termination can be approved or accepted as eligible for reimbursement by the *Community* financial contribution. Any *pre-financing* provided to the *consortium* and any interest generated by the *pre-financing* must be returned in full to the *Commission* within 30 days of notification of termination.

Article 3 - Evolution of the *consortium*

The *consortium* may be enlarged to include other legal entities, which shall accede to the *contract* by means of Form B (set out in Annex V). The *Commission* is deemed to have accepted this legal entity as a *contractor* in the *consortium*, if it does not object within six weeks of receipt of Form B. Any new *contractor* shall comply with the participation rules established by the *Rules for Participation*. This is subject to any condition required by the *Financial Regulation* or other formalities that may be required by any other provision of this *contract*.

They shall assume the rights and obligations of *contractors* as established by the *contract* with effect from the date of their accession to the *contract*. *Contractors* leaving the *consortium* shall be bound by the provisions of the *contract* regarding the terms and conditions applicable to the termination of their participation.

Article 4 - Entry into force of the *contract* and duration of *project*

1. This *contract* shall enter into force on the day of its signature by the *coordinator* and the *Commission*.

2. The duration of the *project* shall be 60 months from 1st February 2006 (hereinafter referred to as the "*start date*").

This *contract* shall be completed once the rights and obligations of all the parties to the *contract* have been met. The implementation and payment phases relating to the *project* must be completed by the *final implementation date* of the *contract*.

The provisions set out in Articles II.7, II.9, II.10, II.11, II.29, II.30, II.31 and Part C of Annex II shall continue to apply after the *final implementation date* as well as any provisions in Annex III which specifically state that they shall continue to apply after the *final implementation date*.

Article 5 - *Community* financial contribution

The *Community* financial contribution shall be in the form of a grant for integration.

The maximum *Community* contribution to the *project* shall be 9,432,600.00 EUR (nine million four

hundred and thirty two thousand six hundred Euro and zero Cents). The *Community* financial contribution shall be limited to the maximum rates of contribution to the activities identified in Part B of Annex II, as modified by any provision of Annex III. Annex I indicates the estimated breakdown of costs and activities to be carried out under the *project*.

Article 6 - Reporting periods

The *project* is divided into reporting periods of the following duration:

- P1: from month 1 to month 12
- P2: from month 13 to month 24
- P3: from month 25 to month 36
- P4: from month 37 to month 48
- P5: from month 49 to the last month of the *project*.

Article 7 - Reports

1. Reports referred to in Article II.7.2 shall be submitted for each reporting period identified in Article 6 within 45 days of the end of the period in question. Reports shall be submitted in English.

2. Reports referred to in Article II.7.3 covering each period shall be submitted at the latest 45 days after the end of each reporting period.

3. In addition to the reports for the last period, final activity and financial reports referred to in Article II. 7.4 (except for the report referred to in Article II.7.4.d)) shall be submitted to the *Commission* at the latest 45 days after the end of the *project*. This delay may be increased by 45 days at the request of the *consortium*. Where the work is completed before the end of the duration of the *project*, the related activity and financial reports shall cover the period up to that date.

Article 8 - Payment modalities

1. The *Community* financial contribution to the *project* shall be paid to the *coordinator* on behalf of the *contractors* in accordance with the following provisions:

a) the *consortium* shall determine the allocation of each tranche of the *Community* financial contribution between the *contractors*, in accordance with this *contract* and any relevant provisions in their *consortium agreement*.

b) the payment of the *Community* financial contribution to the *coordinator* discharges the *Commission* from its obligation to make this payment to the *contractors*.

c) the *coordinator* shall distribute the *Community* financial contribution without unjustified delay. However, the initial *pre-financing* shall not be distributed to the *contractors* until the minimum number of *contractors* required by the *Rules for Participation* have acceded to the *contract*.

2. The *Community* financial contribution shall be paid in accordance with the provisions of Article II.28 and the following:

(a) *pre-financing* of 2,512,804.00 EUR (two million five hundred and twelve thousand eight hundred and four Euro and zero Cents) of the estimated *Community* financial contribution corresponding to the first reporting period and the first six months of the subsequent reporting period indicated in the table of estimated breakdown of costs for this period in Annex I, within 45 days following the date of entry into force of the *contract*.

(b) - within 45 days following approval by the *Commission* of the reports relating to each reporting period:

If an audit certificate has been submitted:

i) a payment to settle the amounts justified and accepted during the reporting period; and

ii) an intermediate *pre-financing* of 85.00% of the estimated *Community* financial contribution corresponding to the subsequent period and the first six months of the period following, indicated in the table of estimated breakdown of costs for this period in Annex I.

Where the amount justified and accepted for the reporting period is less than the *pre-financing* already paid to the *consortium*, that part of the *pre-financing* is re-qualified as a payment and the *Commission* shall deduct the difference from the subsequent *pre-financing*.

Where the amount justified and accepted for the reporting period is more than the *pre-financing* already paid to the *consortium*, the *pre-financing* is re-qualified as a payment and the *Commission* shall add the difference as a complementary payment at the time of the payment of the subsequent *pre-financing*.

If an audit certificate has not been submitted:

i) an intermediate *pre-financing* of 85.00% of the estimated *Community* financial contribution corresponding to the subsequent period and the first six months of the period following, indicated in the table of estimated breakdown of costs for this period in Annex I.

Where the amount justified and accepted for the reporting period is less than the *pre-financing* already paid to the *consortium*, the *Commission* shall deduct the difference from the subsequent *pre-financing*.

Where the amount justified and accepted for the reporting period is more than the *pre-financing* already paid to the *consortium*, the *Commission* shall add the difference to the subsequent *pre-financing*, within the limits established by the *Financial Regulation*.

(c) within 45 days following approval by the *Commission* of the reports relating to the last period and the final reports referred to in Article II.7, the *Commission* shall pay a final payment for that period.

(d) Any payment at the end of a reporting period accompanied by an audit certificate shall be considered as final, subject to the results of any audit or review, which may be carried out pursuant to the provisions of Article II. 29.

Where less than 70% of a *pre-financing* has been used at the end of a reporting period, and notwithstanding the approval by the *Commission* of the related reports, subsequent intermediate pre-financing may be paid only:

(i) if an audit certificate is provided for that reporting period; or

(ii) on the basis of a complementary periodic management report referred to in Article II.7.2 b that shall be submitted to the *Commission* once the above-mentioned spending rate has been achieved.

(e) Where no comments, changes or substantial corrections to any of the *project* activity reports or financial statements are required or where the *Commission* approves the reports more than 45 days after reception, the *Commission* shall make the appropriate payment within 90 days of receipt of the *project* activity reports and associated financial statements.

Where substantial comments, changes, further information or adjustments are requested by the *Commission* within this period, the delay is suspended upon notification by the *Commission*. The remainder of the 90 day payment period begins again only after submission by the *contractors* of the required information.

Article 9 - Special clauses

The following special conditions apply to this *contract*:

1. The contractor(s) shall provide the Commission with a statement confirming that it has received (a) favourable opinion(s) of the relevant ethics committee(s) and, if applicable, the regulatory approval of the competent national authority(ies) in the country concerned before beginning any biomedical research involving human beings.

exists and is made available in the event of an audit.

The *Commission* shall undertake to approve or reject any request for an amendment within 45 days of its receipt. The absence of a response from the *Commission* within 45 days of receipt of such a request, or any other period provided for in the *contract*, does not constitute approval of the request, except for any modification or evolution of the *consortium* as foreseen in Article 3.

All amendments to the *contract* shall be in writing.

Article 11 - Communication

1. Requests for amendments and any communication foreseen by the *contract* shall identify the nature and details of the request or communication and be submitted in writing by means of registered mail with acknowledgement of receipt to the following addresses:

For the *Commission*: Commission of the European Communities
Research Directorate-General
F02
B-1049 Brussels, Belgium

For the *coordinator*: Université Claude Bernard Lyon 1

INSERM U590 - Centre Léon Bérard
28, rue Laënnec,
Lyon, 69008, France

2. Where the *contract* foresees that information or documents are to be transferred by electronic means, the following functional mailboxes shall be used:

For the *Commission*: jan-willem.van-de-loo@cec.eu.int

For the *coordinator*: blay@lyon.fnclcc.fr, jean-yves.blay@chu-lyon.fr

3. The bank account of the *coordinator* to which all payments of the *Community* financial contribution shall be made is:

Name of Account holder: Université Claude Bernard Lyon 1 - Agence comptable
Name of the bank: Trésor Public
IBAN: FR7610071690000000100433072

4. Each party to the *contract* shall inform the other parties without delay of any changes in the names or addresses identified in paragraphs 1 and 2 above.

Article 12 - Applicable law

The law of Belgium shall govern this *contract*.

Article 13 - Jurisdiction

The Court of First Instance or the Court of Justice of the European Communities, as is appropriate in the specific case, shall have sole jurisdiction to hear any disputes between the *Community* and the *contractors* as regards the validity, the application or any interpretation of this *contract*.

Article 14 - Annexes forming an integral part of this contract:

1. The following annexes form an integral part of this *contract*:

Annex I - Description of work
Annex II - General Conditions
Annex III - Specific provisions related to Network of Excellence
Annex IV - Form A - consent of *contractors* to accede to the *contract*
Annex V - Form B - accession of new legal entities to the *contract*
Annex VI - Form C - financial statement per instrument

2. In the event of any conflict between the provisions of the Annexes to this *contract* and any provision of this part of the *contract*, the latter shall take precedence. The provisions of Annex III shall take precedence over the provisions of Annex II, and both shall take precedence over the provisions of Annex I.

3. The special conditions set out in Article 9 shall take precedence over any other provisions of this *contract*.

FORM A - ACCESSION TO THE CONTRACT

Università Degli Studi di Padova established in Italy, Via VIII Febbraio , 35128, Padova represented by Mario Lise, Director, and/or Alberto Amadori, Vice-director, or her/his/their authorised representative, hereby consents to become a *contractor* to *contract* number **018806** (relating to *project* **"CONNective TIssue CAncers NETwork to integrate European Experience in Adults and Children"**) signed between the **Commission of the European Communities** (the "*Commission*") and **Université Claude Bernard Lyon 1** established in France, 43 bd du 11 novembre 1918, 69622, Lyon (the "*coordinator*"), and accepts in accordance with the provisions of the aforementioned *contract* all the rights and obligations of a *contractor*.

Done in 3 copies, of which one shall be kept by the *coordinator* and one by **Università Degli Studi di Padova**, the third being sent to the *Commission* by the *coordinator* in accordance with Article 2.1 and Article 11 of the *contract*.

For the contractor
Università Degli Studi di Padova

Prof. Mario Lise
Name of the first legally authorised representative

DIRECTOR
Function of the first legally authorised representative

[signature]
Signature of the first legally authorised representative

Prof. Alberto Amadori
Name of the second legally authorised representative

VICE - DIRECTOR
Function of the second legally authorised representative

[signature]
Signature of the second legally authorised representative

15 DIC 2005
Date
(stamp or seal of the organisation)

UNIVERSITA' DI PADOVA
DIPARTIMENTO DI SCIENZE ONCOLOGICHE E CHIRURGICHE
Via Gattamelata, 64 Tel. 8215644
35128 PADOVA

For the coordinator
Université Claude Bernard Lyon 1

DOMINIQUE DEBOUZIE
Name of the first legally authorised representative

PRESIDENT
Function of the first legally authorised representative

[signature]
Signature of the first legally authorised representative

Name of the second legally authorised representative

Function of the second legally authorised representative

Signature of the second legally authorised representative

09.02.06
Date
(stamp or seal of the organisation)
