# Supplementary material for: A European project on incidence, treatment, and outcome of sarcoma
Source: BMC Public Health. 2010 Apr 12;10:188. doi: 10.1186/1471-2458-10-188 (PMC2882909; doi:10.1186/1471-2458-10-188)
Supplement: Additional file 2 — WHO Classification of Sarcomas. [file 1471-2458-10-188-S2.DOCX]

# Appendix 1. WHO Classification of Sarcomas

| **Histotype** | **SNOMED** |
| --- | --- |
| Liposarcoma - Well Differentiated | M-88513 |
| Liposarcoma – Dedifferentiated | M-88583 |
| Liposarcoma - Myxoid | M-88523 |
| Liposarcoma - Round Cell | M-88533 |
| Liposarcoma - Mixed Type | M-88553 |
| Liposarcoma - Pleomorphic | M-88543 |
| Liposarcoma - NOS | M-88503 |
| Leiomyosarcoma | M-88903 |
| Synovial Sarcoma - Monophasic | M-90413 |
| Synovial Sarcoma - Biphasic | M-90433 |
| Synovial Sarcoma - Poorly Differentiated | M-90403 |
| Malignant Peripheral Sheath Tumor | M-95403 |
| Myxofibrosarcoma | M-88113 |
| Pleomorphic Sarcoma / MFH | M-88303 |
| Fibrosarcoma | M-88103 |
| Congenital Fibrosarcoma | M-88143 |
| Dermatofibrosarcoma Protuberans | M-88323 |
| Epithelioid Sarcoma | M-88403 |
| Alveolar Soft Part Sarcoma | M-95813 |
| Clear Cell Sarcoma | M-90443 |
| Malignant Mesenchymoma | M-89903 |
| Malignant Solitary Fibrous Tumor / Haemangiopericytoma | M-88153 |
| Low Grade Myofibroblastic Sarcoma | M-88253 |
| Myxoinflammatory Fobroblastic Sarcoma | M-88113 |
| Low Grade Fibromyxoid Sarcoma | M-88113 |
| Sclerosing Epithelioid Fibrosarcoma | M-88103 |
| Embryonal Rhabdomyosarcoma | M-89103 |
| Alveolar Rhabdomyosarcoma | M-89203 |
| Pleomorphic Rhabdomyosarcoma | M-89013 |
| Spindle Cell Rhabdomyosarcoma | M-89123 |
| Rhabdomyosarcoma - NOS | M-89003 |
| Epithelioid Haemangioendothelioma | M-91333 |
| Angiosarcoma | M-91203 |
| Kaposi Sarcoma | M-91403 |
| Extraskeletal Myxoid Chondrosarcoma | M-92313 |
| Mesenchymal Chondrosarcoma | M-92403 |
| Chondrosarcoma NOS | M-92203 |
| Extraskeletal Osteosarcoma | M-91803 |
| Ewing / Pnet | M-92603 |
| Extrarenal Rhabdoid Tumor | M-89633 |
| Desmoplastic Round Cell Tumor | M-88063 |
| Intimal Sarcoma | M-88003 |
| Other Sarcoma - Sarcoma NOS | M-88003 |
